# Correction: Mitochondrial morphodynamics alteration induced by influenza virus infection as a new antiviral strategy

**DOI:** 10.1371/journal.ppat.1009485

**Published:** 2021-03-31

**Authors:** Irene Pila-Castellanos, Diana Molino, Joe McKellar, Laetitia Lines, Juliane Da Graca, Marine Tauziet, Laurent Chanteloup, Ivan Mikaelian, Laurène Meyniel-Schicklin, Patrice Codogno, Jacky Vonderscher, Cédric Delevoye, Olivier Moncorgé, Eric Meldrum, Caroline Goujon, Etienne Morel, Benoit de Chassey

There is an error in affiliation designation for authors Caroline Goujon and Olivier Moncorgé. The correct affiliation is affiliation 3: Institut de Recherche en Infectiologie de Montpellier (IRIM), UMR 9004—CNRS, Université de Montpellier, Montpellier, France.
