# Supplementary figures and images for: Mitochondrial morphodynamics alteration induced by influenza virus infection as a new antiviral strategy
Source: PLoS Pathog. 2021 Feb 17;17(2):e1009340. doi: 10.1371/journal.ppat.1009340 (PMC7920353; doi:10.1371/journal.ppat.1009340)

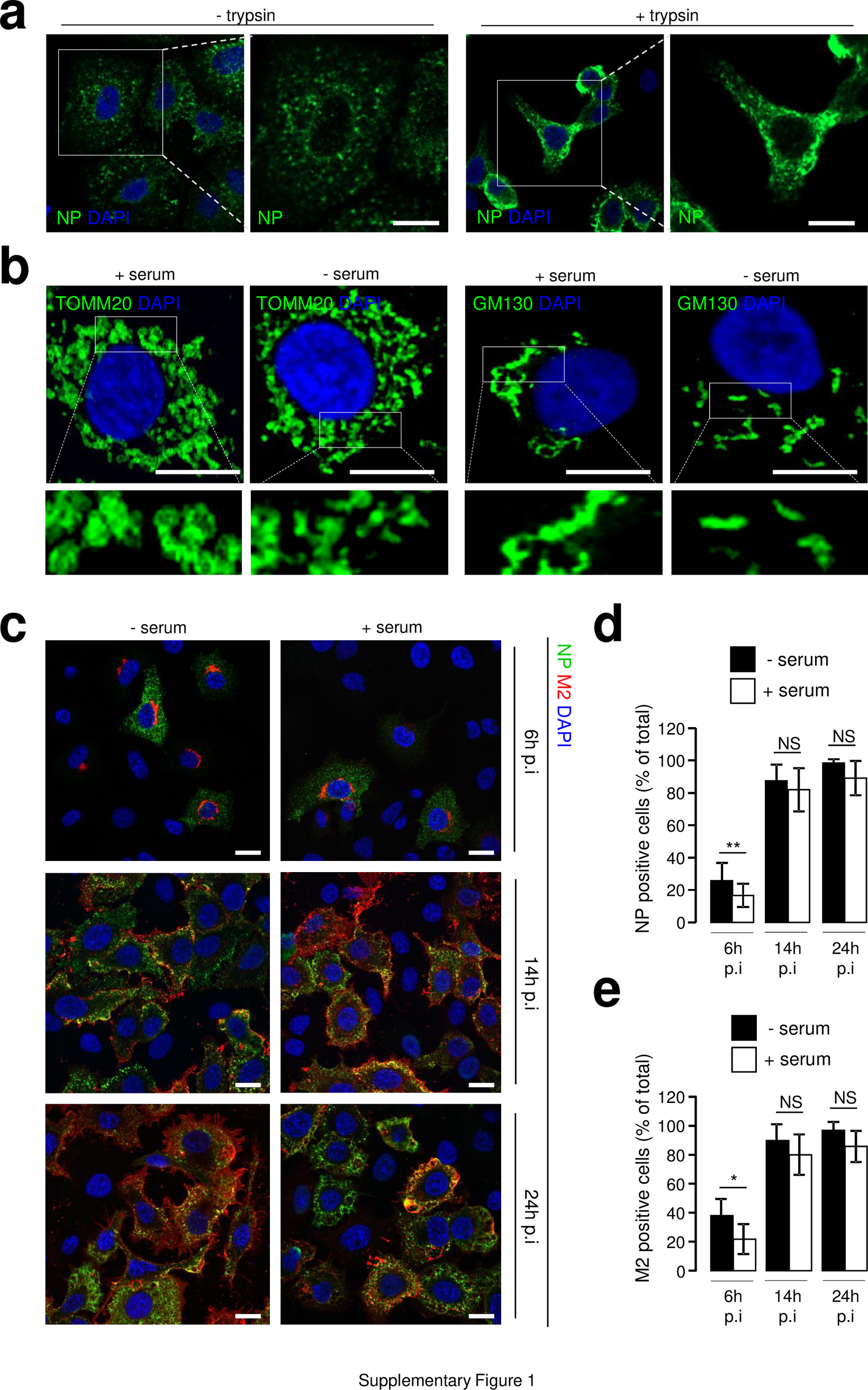

Supplement: S1 Fig — a, A549 cells infected with influenza H1N1 virus at MOI 1 for 24h without serum and treated with trypsin (or not) were immunostained with anti-NP antibody (green) and DAPI (blue). b, A549 cells cultured with serum (or not) for 24h were immunostained with anti-TOMM20 antibody (green), DAPI (blue, left panel) and anti-GM130 antibody (green) and DAPI (blue, right panel); cropped areas show mitochondria and Golgi morphology, modified upon serum starvation. c, A549 cells were infected with H1N1 virus at MOI 1 in serum-free medium supplemented with trypsin. After 1h, cells were washed, and the medium was replaced (or not) with medium containing serum. Cells were immunostained after 6h, 14h and 24h post infection with anti-NP antibody (green), anti-M2 antibody (red) and DAPI (blue). d, Quantification of infected cells (NP signal) from single cells illustrated in (c). e, Quantification of infected cells (M2 signal) from single cells illustrated in (c). Scale bars = 10μm. For evaluating significance of differences observed in d and e two-tailed Student’s t test was used (** indicates p<0.001, NS for non-significant). (TIF) [file ppat.1009340.s001.tif]

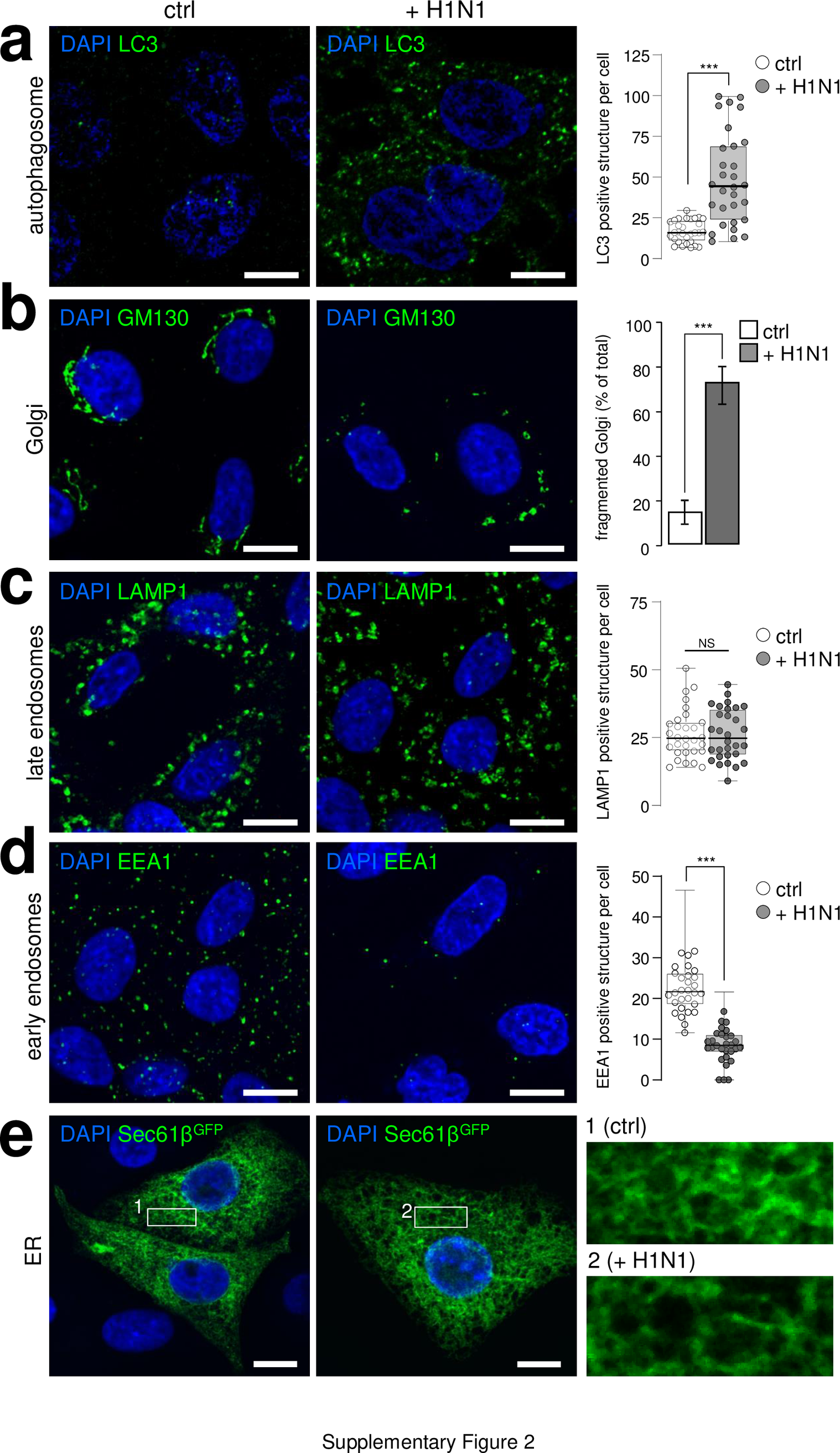

Supplement: S2 Fig — a, A549 cells infected (or not) with H1N1 virus at MOI 1 for 24h were immunostained with anti-LC3 antibody (green) and DAPI (blue) and pictures were quantified for LC3 positive structures per cell (N = 30 cells from three independent experiments). b, A549 cells infected (or not) with H1N1 virus at MOI 1 for 24h were immunostained with anti-GM130 antibody (green) and DAPI (blue) and pictures were quantified for fragmented Golgi from single cells (N = 30 cells from three independent experiments). c, A549 cells infected (or not) with H1N1 virus at MOI 1 for 24h were immunostained with anti-LAMP1 antibody (green) and DAPI (blue) and pictures were quantified for LAMP1 positive structures per cell (N = 30 cells from three independent experiments). d, A549 cells infected (or not) with H1N1 virus at MOI 1 for 24h were immunostained with anti-EEA1 antibody (green) and DAPI (blue) and pictures were quantified for EEA1 positive structure per cell (N = 30 cells from three independent experiments). e, A549-Sec61β-GFP stable cell line was infected (or not) with H1N1 virus at MOI 1 for 24h and immunostained with DAPI (blue); cropped areas show ER morphology (N = 30 cells from three independent experiments). Scale bars = 10μm. For evaluating significance of differences observed in a, b, c and d, a two-tailed Student’s t test was used (*** indicates p<0.0001, NS for non-significant). (TIF) [file ppat.1009340.s002.tif]

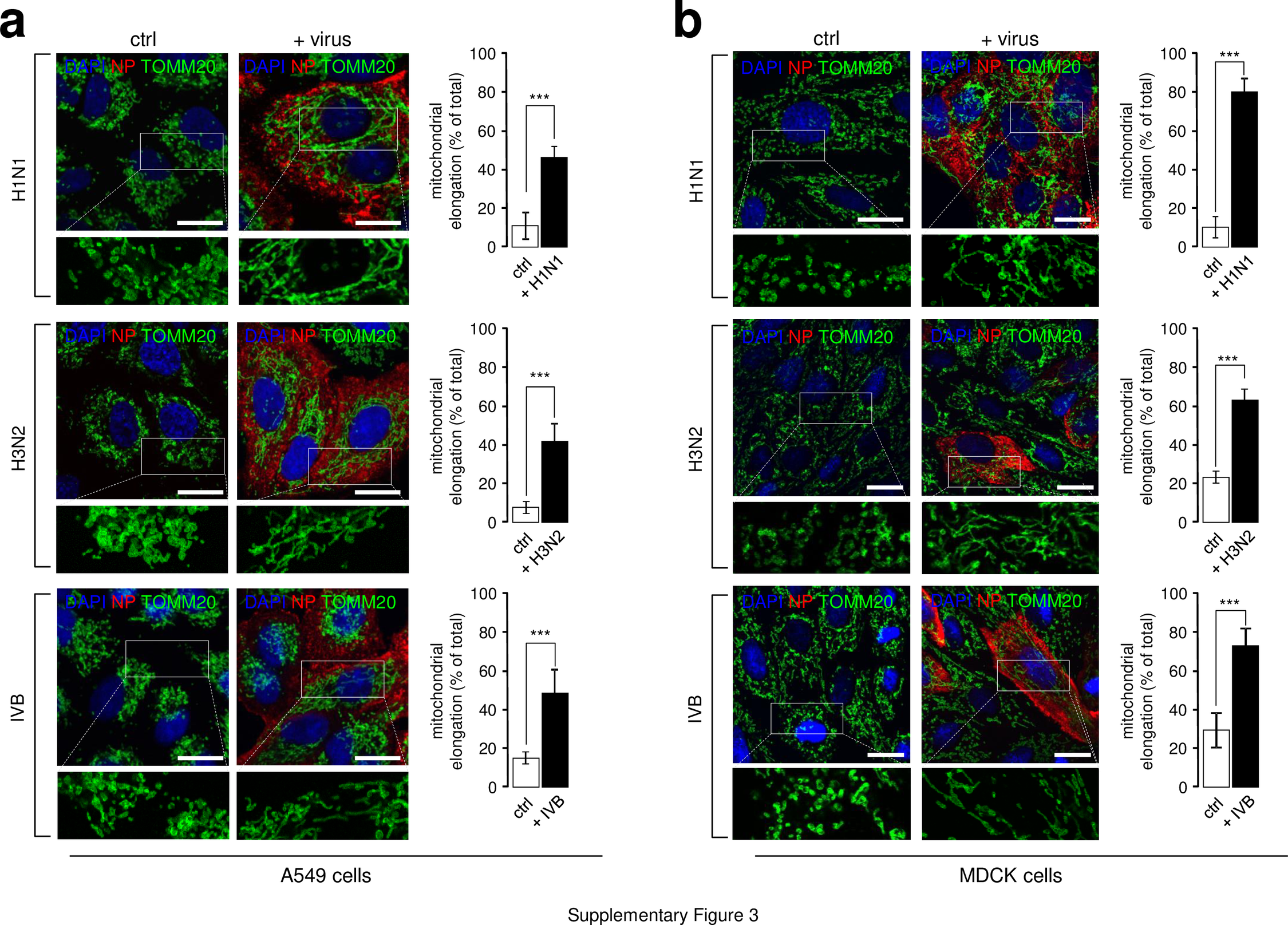

Supplement: S3 Fig — a, A549 cells infected (or not) with influenza A H1N1, influenza A H3N2 or influenza B (IBV) viruses at MOI 1 for 24h were immunostained with anti-TOMM20 antibody (green), anti-NP antibody (red) and DAPI (blue) and pictures were quantified for mitochondrial elongation from single cells (N = 50 cells from three independent experiments); cropped areas show mitochondria morphology. b, MDCK cells infected (or not) with influenza A H1N1, influenza A H3N2 or IBV viruses at MOI 1 for 24h were immunostained with anti-TOMM20 antibody (green), anti-NP antibody (red) and DAPI (blue) and pictures were quantified for mitochondrial elongation from single cells (N = 50 cells from three independent experiments); cropped areas show mitochondria morphology; cropped areas show mitochondria morphology. Scale bars = 10μm. For evaluating significance of differences observed in a and b two-tailed Student’s t test was used (*** indicates p<0.0001; NS for non-significant). (TIF) [file ppat.1009340.s003.tif]

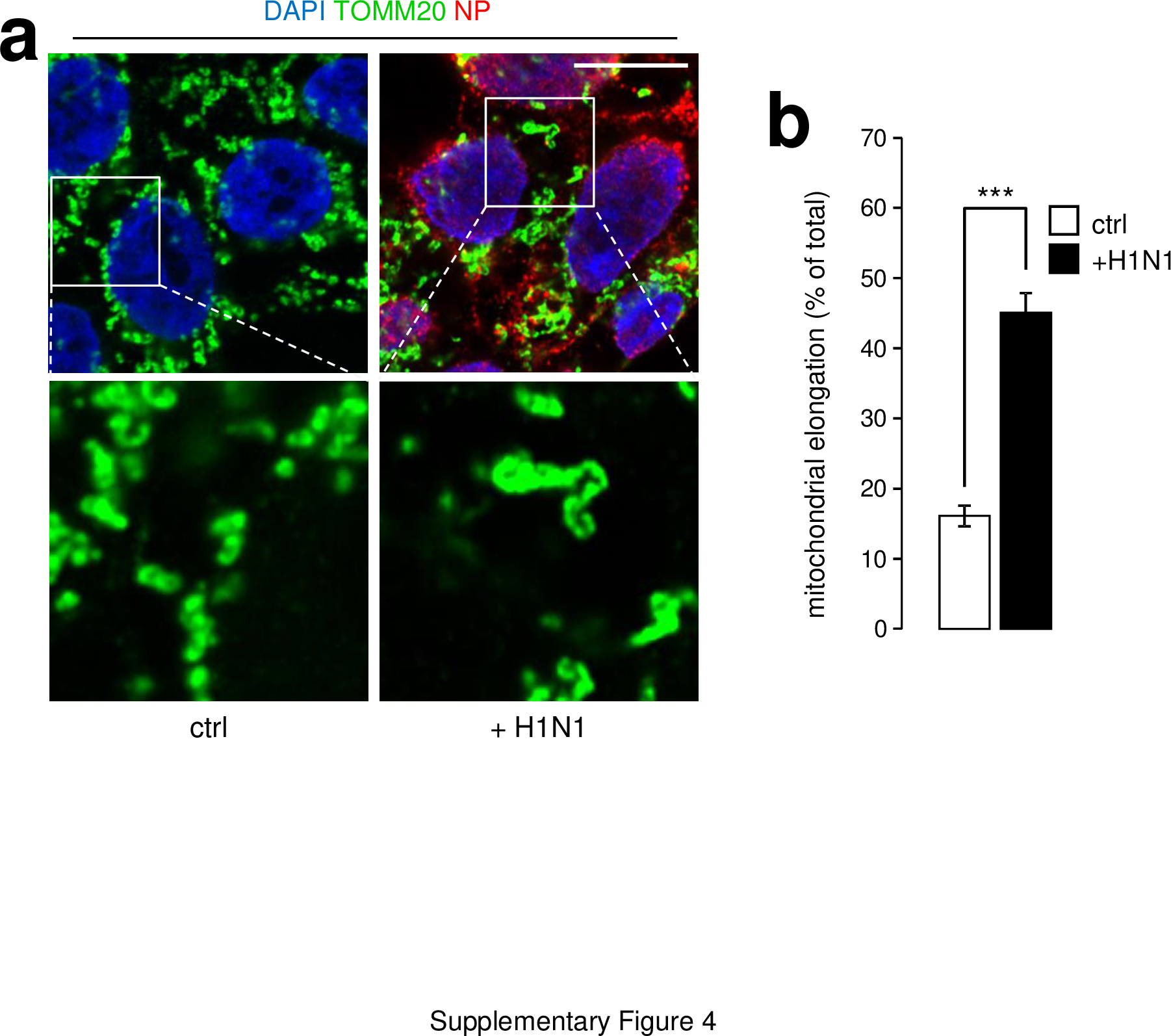

Supplement: S4 Fig — a, HEK293T cells infected (or not) with influenza A H1N1 at MOI 1 for 15h were immunostained with anti-TOMM20 antibody (green), anti-NP antibody (red) and DAPI (blue) b, Pictures were quantified for mitochondrial elongation from single cells (N = 50 cells from three independent experiments); cropped areas show mitochondria morphology. Scale bars = 10μm. For evaluating significance of differences observed in a and b two-tailed Student’s t test was used (*** indicates p<0.0001; NS for non-significant). (TIF) [file ppat.1009340.s004.tif]

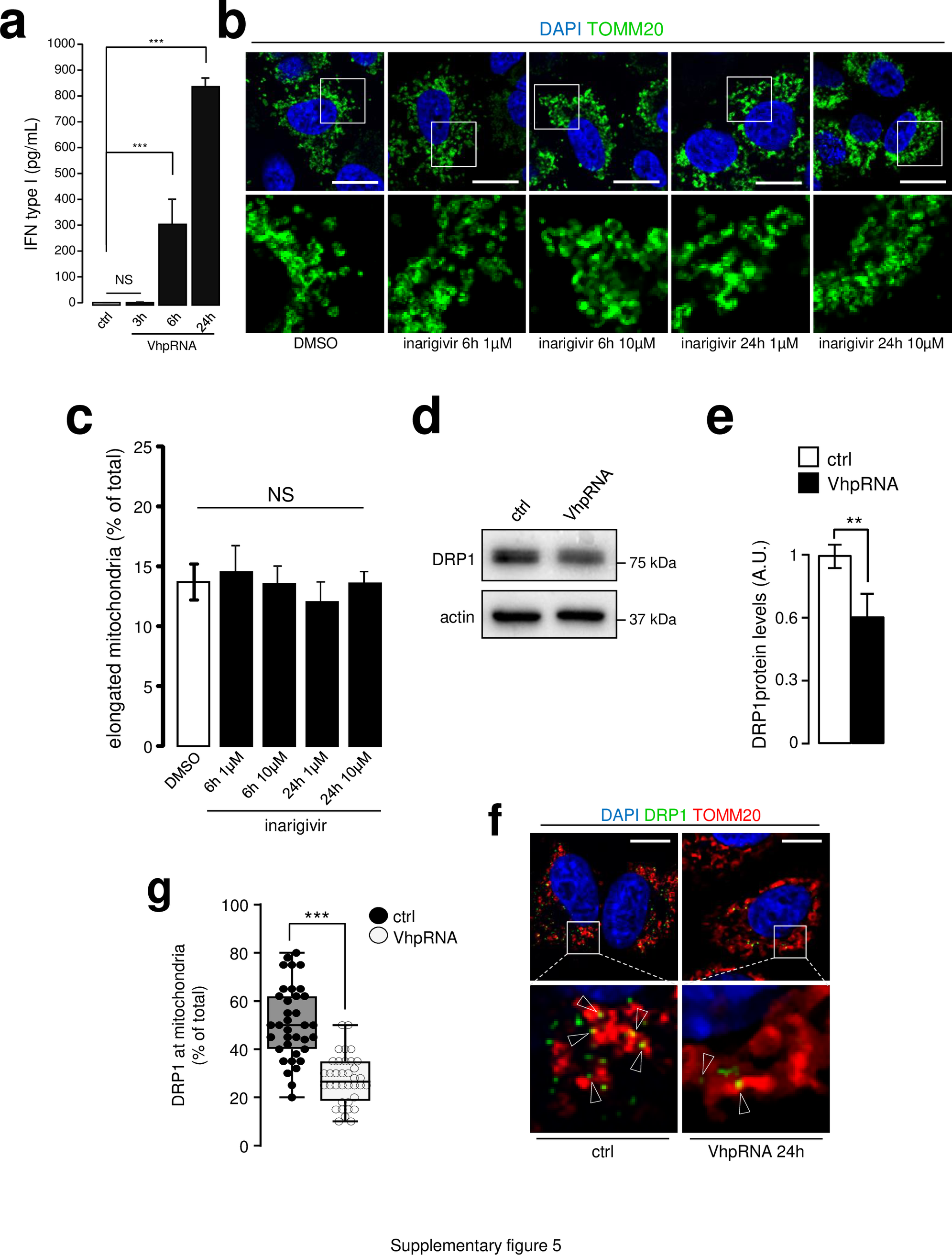

Supplement: S5 Fig — a, A549 cells were transfected (or not) by VhpRNA for 3h, 6h and 24h and IFNβ secretion was measured in cell supernatant (N = 3). b, A549 cells were treated (or not) by 1μM or 10μM Inarigivir for 6h and 24h and immunostained with anti-TOMM20 antibody (green) and DAPI. (blue). Cropped areas show mitochondria morphology. Scale bars = 10μm. For evaluating significance of differences observed in a and b two-tailed Student’s t test was used (*** indicates p<0.0001; NS for non-significant).c, Pictures exemplified in b were quantified for mitochondrial elongation from single cells (N = 50 cells from three independent experiments). d, Representative western blot analysis of DRP1 in A549 cells transfected (or not), with VhpRNA, 24h post-transfection. e, Quantification of DRP1 western blots as showed in (c) (N = 4). f, A549 cells transfected (or mock transfected) with VhpRNA were immune-stained with anti-DRP1 antibody (green), anti-TOMM20 antibody (red) and DAPI (blue); cropped areas show DRP1 at the mitochondria (N = 3). g, Quantification of DRP1 signal on TOMM20 positive structures from A549 cells illustrated in (i) (N = 30 cells from three independent experiments). For evaluating significance of differences observed in a, c, e and g, a two-tailed Student’s t test was used (*** indicates p<0.0001). (TIF) [file ppat.1009340.s005.tif]

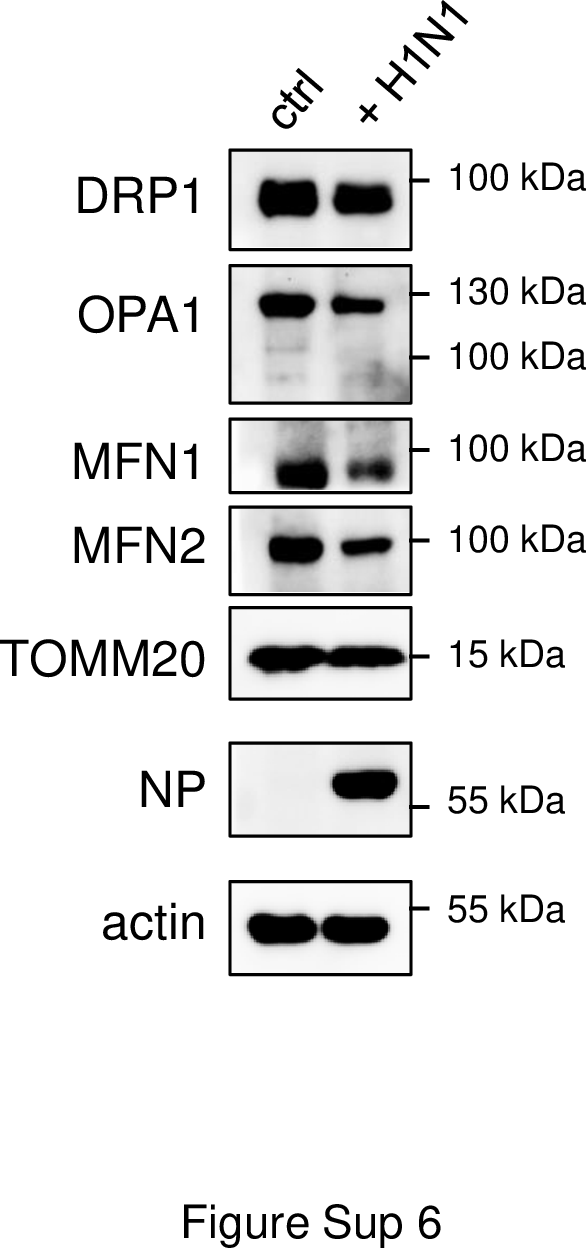

Supplement: S6 Fig — a, Representative western blot analysis of DRP1, OPA1, MFN1, MFN2, TOMM20, NP and actin in A549 cells infected (or not), with influenza A H1N1 virus at MOI 1 for 24h. (TIF) [file ppat.1009340.s006.tif]

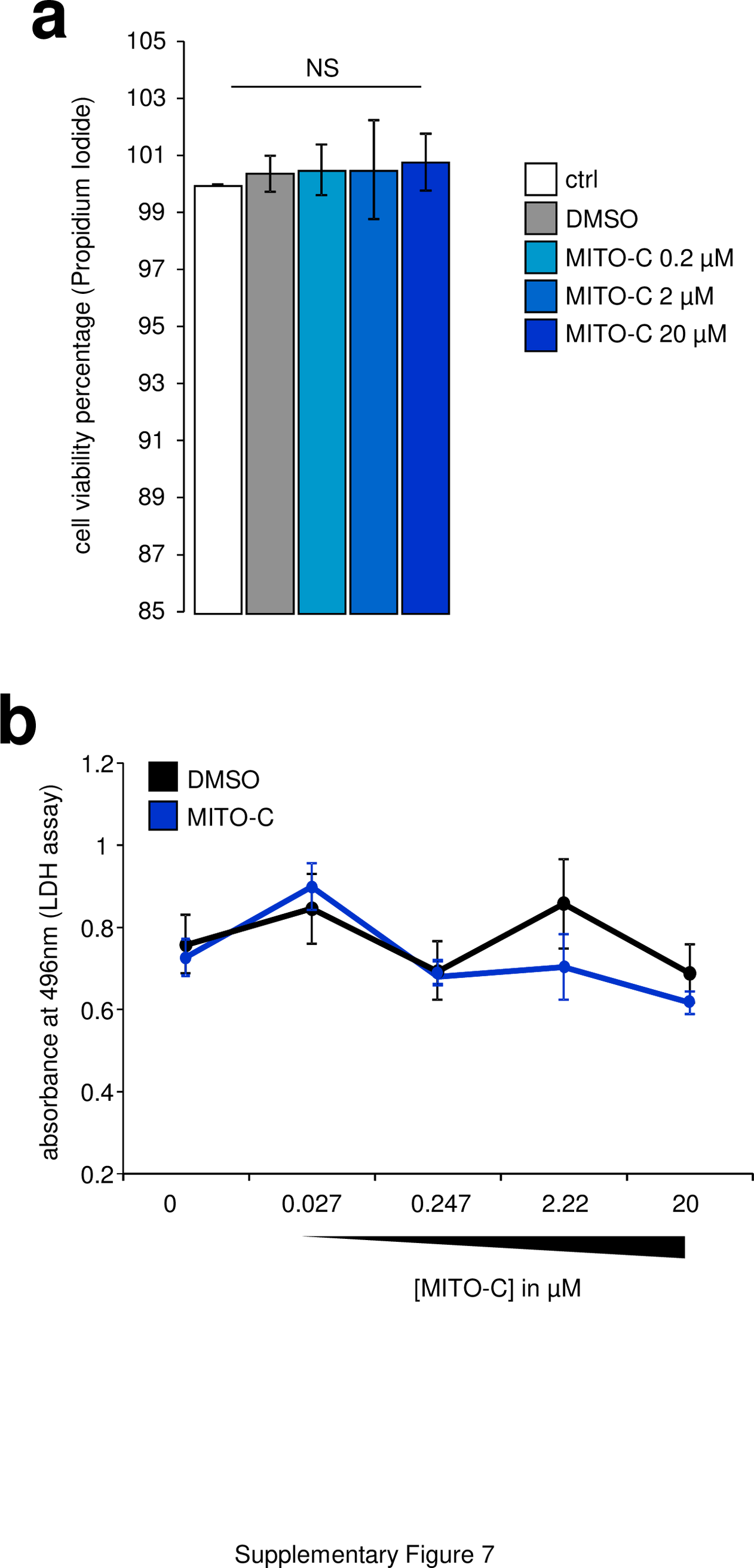

Supplement: S7 Fig — a, A549 cells treated for 48h with increasing and indicated concentrations of Mito-C, DMSO (vehicle) or culture media without vehicle were stained with propidium iodide (PI) and analyzed by cytometry (N = 3). b, Lactate dehydrogenase (LDH) enzyme release was measured in supernatants of A549 cells treated for 48h with increasing concentrations of Mito-C or DMSO (N = 3). For evaluating significance of differences observed in a and b, a two-tailed Student’s t test (NS for non-significant). (TIF) [file ppat.1009340.s007.tif]

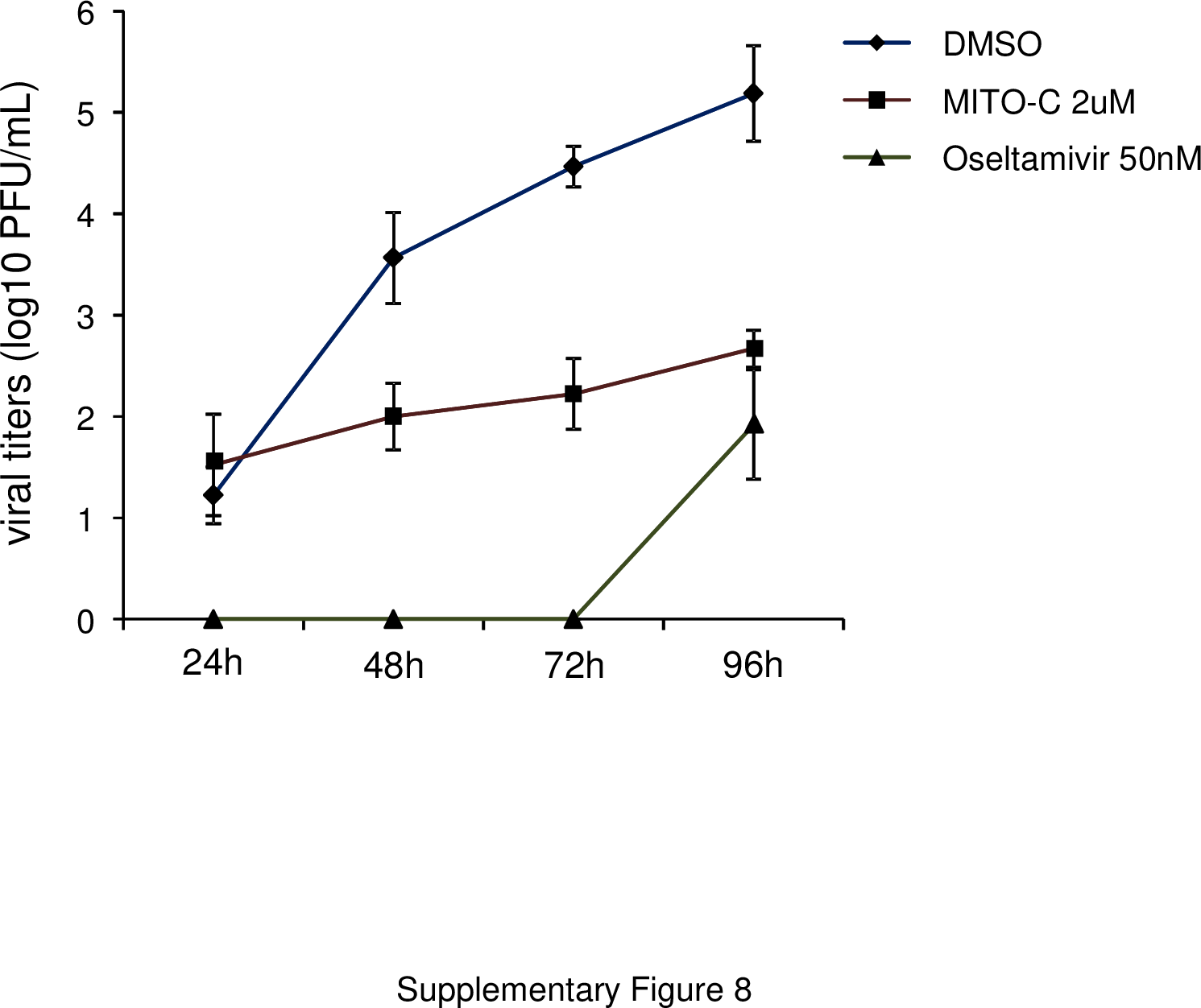

Supplement: S8 Fig — a, A549 cells were infected at an MOI of 0.005 PFU/cell with A/Victoria/3/75 virus and treated with Mito-C (2μM), Oseltamivir (50nM) or DMSO. At the indicated times post-infection, viral titers were determined by standard plaque assay on MDCK cells. The results are shown as the mean ± SD of three independent experiments. (TIF) [file ppat.1009340.s008.tif]

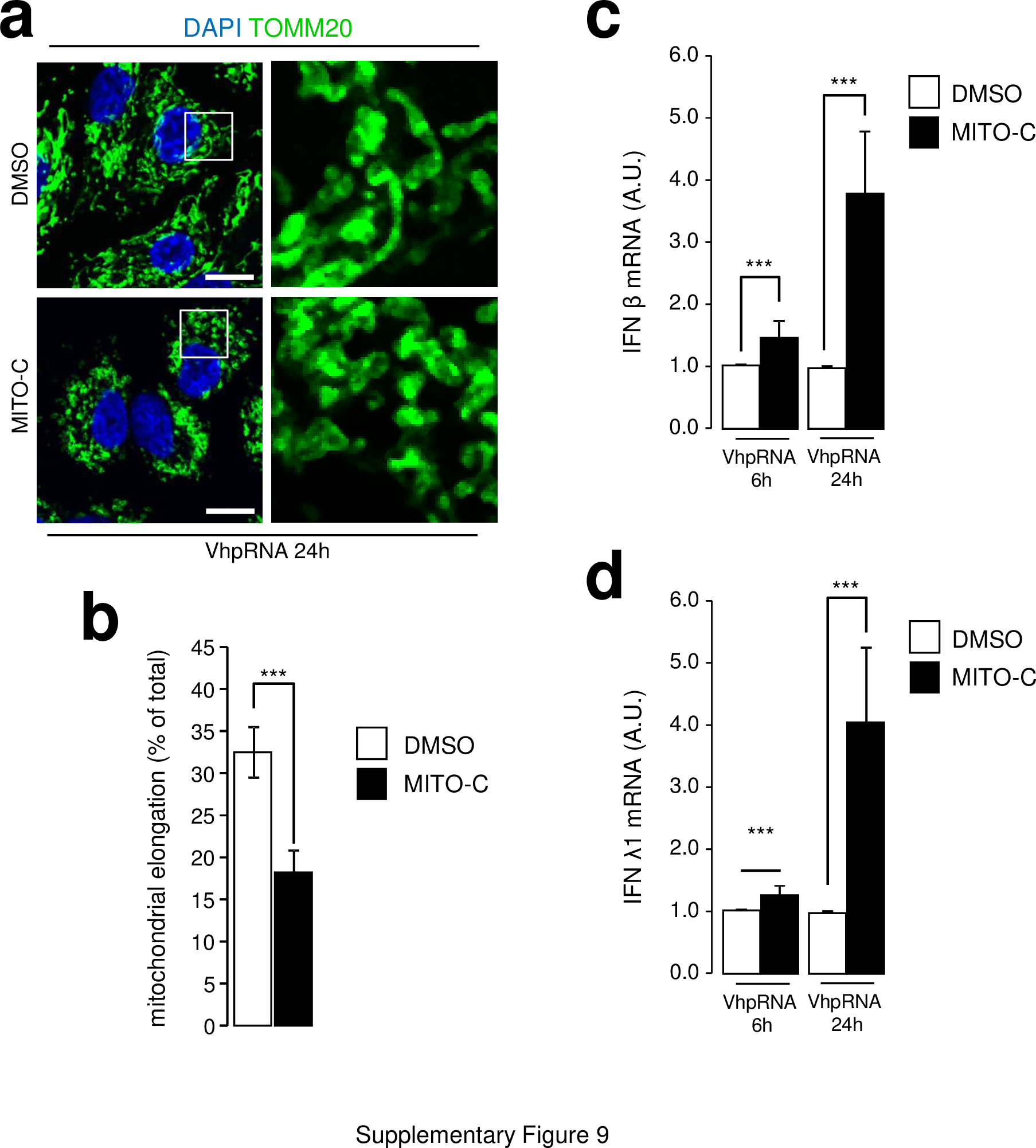

Supplement: S9 Fig — a, A549 cells, transfected with vh-RNA and treated with Mito-C at 2μM or DMSO, were immunostained with anti-TOMM20 antibody (green) and DAPI (blue) 24h post-transfection. All scale bars = 10μm. b, Quantification of mitochondrial elongation (TOMM20 signal) from single cells illustrated in (a) (N = 50 cells from three independent experiments) c, RT-qPCR analysis of IFNλ1 mRNA from A549 cells transfected (or mock transfected) with vh-RNA and treated with Mito-C at 2μM or with DMSO, at 6h or 24h post transfection (N = 3). d, RT-qPCR analysis of IFNβ mRNA from A549 cells transfected (or mock transfected) with vh-RNA and treated with Mito-C at 2μM or with DMSO, at 6h or 24h post transfection (N = 3For evaluating significance of differences observed in c, e, g, i, k and l, two-tailed Student’s t test was used (*** indicates p<0.0001). (TIF) [file ppat.1009340.s009.tif]
