# Supplementary material for: Mitochondrial morphodynamics alteration induced by influenza virus infection as a new antiviral strategy
Source: PLoS Pathog. 2021 Feb 17;17(2):e1009340. doi: 10.1371/journal.ppat.1009340 (PMC7920353; doi:10.1371/journal.ppat.1009340)
Supplement: Attachment — Submitted filename: Response to reviewers.docx [file ppat.1009340.s010.docx]

**Pila et al./Response to reviewers**

We wish to thank the three reviewers for their useful and precise comments which have considerably improved the quality of the manuscript and have strengthened our conclusions. We hope that you will share our excitement for this revised manuscript and consider it for publication in PLoS Pathogens.

**Part II – Major Issues: Key Experiments Required for Acceptance**

Reviewer #1: This paper is interesting and the conclusions raised by the authors are well supported by the data.
My only concern is about the fact that the authors report, based on IF imaging, that the influenza infection seems to induce a strong mitochondria-to-cytosol relocalization of DRP1. Unfortunately, this observation can not be confirmed by WB after cell fractionation because the viral infection surprisingly promotes a degradation of DRP1. Based on their results, it appears that the RIG-I-MAVS pathway regulate the mitochondrial dynamics. How do the authors explain that the RIG-I-MAVS pathway controls the DRP1 stability at the mitochondria? Does DRP1 undergo post-translational modification(s) in the context of influenza infection? In agreement with a previous report (PMID: 19066474), the authors observed an increase in autophagy after infection, so is it possible that DRP1 is degraded through autophagy as described recently for MAVS (PMID: 31304625)? If it is not due to autophagy, does a proteasome inhibition prevent this DRP1 degradation?

We thank this reviewer for this interesting question. Autophagy and proteasome inhibition are both described to inhibit influenza virus replication (#19066474, #22286278, #22900043, #20592098, #24669782). Therefore, the use of inhibitors of proteasome or autophagy will also inhibit mitochondria elongation associated with virus replication. The consequence of inhibiting proteasome or autophagy would then be an inhibition of DRP1 amount modulation due to inhibition of virus replication and not as a direct consequence on DRP1 stability. One way to answer to this reviewer’s comment is to test inhibitors for proteasomal and autophagosomal degradation(s) in the context of viral RNA transfection, which mimics (as notably shown in the present paper) the H1N1 infection on mitochondria morphodynamics.

For this purpose, we first validated the viral hairpin RNA (VhpRNA) transfection as a surrogate of H1N1 viral infection on the three main phenotypes induced by the virus and highlighted in the paper: mitochondria elongation, reduced expression of DRP1 at the mitochondria and mitochondria-ER contact sites reduction. We have already demonstrated that similar to the virus, the transfection of viral hairpin RNA induces mitochondria elongation (previous supplementary figure 4b and 4c, **now figure 2a, 2b**). In follow up experiment, we now also show that DRP1 protein stability at the mitochondria is reduced in these conditions (**supplementary figure 5f and 5g**). In addition, confocal microscopy experiments show that the number of ER-mitochondria contact sites (notably identified with an anti-PTPIP51 antibody by immunofluorescence) is significantly reduced after VhpRNA 24h post-transfection (**Figure 2f, 2g**). Electron microscopy experiments consistently confirmed the reduction of the number of contact sites (**Figure 2c, 2d and 2e,**). Overall, these data validate the ability of the viral hairpin RNA transfection to mimic influenza infection specifically addressing the question of the first reviewer. The manuscript has been modified accordingly.

As suggested by this reviewer, we used pharmacological compounds such as MG132 and 3MA (3-methyladenine) drugs to inhibit the proteasome and autophagy, respectively (#8087844, #6952238). Without viral hairpin RNA transfection these molecules do not modify mitochondria morphology neither 6h nor 24h post-treatment (Reviewer 1, **rebuttal figure 1a**). Importantly, MG132 and 3MA have no impact on the elongation of mitochondria induced by VhpRNA transfection (Reviewer 1, **rebuttal figure 1a**). Interestingly, while these drugs inhibit DRP1 degradation under viral hairpin RNA transfection (Reviewer1, **rebuttal figure 1b**), they increase the presence of DRP1 on the mitochondria (Reviewer 1, **rebuttal figure 1c**). With these data in mind, it appears that beyond the stability of DRP1 at the mitochondrial membrane, additional mechanistic events are required for the mitochondrial morphodynamics regulation reported in our manuscript. This could notably be associated with a specific contribution of a mitochondrial interactor of DRP1 and as suggested by this reviewer a post-translational modification of DRP1.

***Reviewer 1, Rebuttal Figure 1. a****, A549 cells transfected (or not) with dsRNA for 24h were treated (or not) with  1mM 3-MA or 10µM MG-132 for 24h and immunostained with anti-TOMM20 antibody (green) and DAPI (blue); cropped areas show mitochondria morphology, elongated in transfected condition (N=3).* ***b****, Representative western blot analysis of DRP1 in A549 cells transfected (or not) with vh-RNA and treated (or not) with 1mM 3-MA or 10µM MG-132 for 24h.* ***c****, A549 cells transfected (or not) with dsRNA for 24h were treated (or not) with  1mM 3-MA or 10µM MG-132 for 24h and immunostained with anti-DRP1 antibody (green), anti-TOMM20 antibody (red) and DAPI (blue); cropped areas show DRP1 at the mitochondria (N=3).*

Importantly, during the revision of our manuscript, Chen et al. (#33171123) published interesting insights to this precise question. Following innate RNA sensing through RIG-I-like receptors, the TBK1 kinase phosphorylates directly, and massively, DRP1 at residues S412 and S684. This phosphorylation prevents DRP1 oligomerization and DRP1 aggregation to the mitochondria when overexpressed in cells and thus abrogates the mitochondrial fission activity of DRP1. This article is now mentioned in the introduction.

Strikingly, the above-mentioned additional experiments suggest that the simple stability/presence of DRP1 on the mitochondria is not sufficient to induce mitochondrial fission. Thus, it would be interesting in future experiments to test the phosphorylation status of DRP1 (S412 and S684) during influenza virus infection and, as we did here, in the presence of Vhp RNA in cells treated or not with MG132 or 3MA. Despite its inability to induce fission, phospho-DRP1 could be retained on the mitochondria by interacting with additional molecules, the degradation of which would be prevented by proteasome or autophagy inhibition. This is now mentioned in the discussion.

In figure 2E, the infection increased OPA1 detection and the authors propose that it is likely a consequence of a coalescence of this protein in the mitochondria. In agreement, OPA1 aggregation have been demonstrated to be required for its pro-fusion activity (PMID: 31292547). This may be mention in the text. Have the authors investigated by WB the different isoforms of OPA1 during infection?

We followed the recommendation of this reviewer (that matches **the 2.2 major point of reviewer #2**) and we now present a western blot for OPA1 expression (**Supplementary Figure 6**). In the given conditions, we did not detect any change in OPA1 isoforms following infection. The long isoform (L-OPA1) remains the main isoform. This isoform has been previously associated with fusion (#17003040, #27189080) while shorter forms (S-OPA1) are considered fission mediators (#24616225). The L-OPA1 vs S-OPA1 ratio is thus still in favor of a potential fusion and this is thus consistent with our experimental data.

Surprisingly, the total amount of OPA1 is slightly reduced following infection while the mitochondrial mass is not particularly modified as observed through TOM20 expression (**Supplementary Figure 6**). Importantly and worth notably, OPA1 is a protein with a high propensity for aggregation. During infection, we demonstrate in our experiments that the protein coalesces at the mitochondria membrane. It is likely that the coalesced and aggregated OPA1 may escape the cellular lysis.

Reviewer #2: 1. The authors found that influenza A viral RNA could be a stimulator to induce mitochondrial elongation. The result also suggested that stimulation of RIG-I signaling pathway could cause this phenotype.
(1) Does the viral RNA also reduce the contact sites of Mito/ER, like the H1N1 infection?

We thank this reviewer for this very important question.

We addressed this point through two different electron and light microscopy-based experiments. Confocal microscopy analyzes first show that the number of contact sites (as notably determined by PTPIP51 ER-mito interface protein staining) is significantly reduced 24h post-transfection of the viral hairpin RNA (VhpRNA, **figure 2f, 2g**). Electron microscopy experiments consistently confirmed the reduction of the number of contact sites at the ultrastructural level (**figure 2c, 2d, 2e**). We further show that DRP1 protein stability at the mitochondrial membrane is reduced as well in these conditions (supplementary figure 5f and 5g). Overall, we report that the transfection of VhpRNA in A549 cells recapitulates three phenotypes associated with H1N1 infection: mitochondria elongation, reduced expression of DRP1 on the mitochondria and ER-mitochondria contact sites reduction. The manuscript has been modified accordingly.

Interestingly, this result echoes a recent study that has been published during the revision of this article showing that RNA sensing through RIG-I-like receptors induces mitochondrial elongation (#33171123). We found no reference to a modulation of ER-mitochondria contact sites in this article. Our results complement this recently published observation and opens information for future mechanistic studies.

(2) In the experiments, the authors used 87-mer 5’ triphosphated hairpin RNA derived from an H1N1 virus. The authors should describe the sequence of the 87-mer RNA in the Materials and Methods section.

The sequence is actually a 89-mer RNA. This typo has been modified in the Materials and Methods. The sequence given by InvivoGen is now described in this section:

5’pppGGAGCAAAAGCAGGGUGACAAAGACAUAAUGGAUCCAAACACUGUGUCAAGCUUUCAGGUAGAUUGCUUUCUUUGGCAUGUCCGCAAAC- 3’

2. The results showed that infection of H1N1 virus relocalized DRP1 and decreased DRP1 expression, whereas the OPA1 signal at mitochondria was increased in the infected cells.
(1) The authors should show a blot for at least one viral protein in Fig. 2C.

We agree with this reviewer that since we present western blot data in normal and infected conditions, we must illustrate that the lysates are from infected cells or not. We apologize for this omission.

The western blot showing the expression of the viral nucleoprotein (NP) is now shown in **supplementary figure 6**.

(2) Is the expression of OPA1 changed in the infected cells? The authors may demonstrate it by a Western blot.

We followed the recommendation of this reviewer that matched the 2nd major point of reviewer #1 and we now present a western blot for OPA1 expression (**Supplementary Figure 6**). We did not detect any change in OPA1 isoforms following infection. The long isoform (L-OPA1) remains the main isoform. This isoform has been previously associated with fusion (#17003040, #27189080) while the shorter form (S-OPA1) is considered fission mediators (#24616225). The L-OPA1 versus S-OPA1 ratio is still in favor of a potential fusion and consistent with our experimental data.

Surprisingly, the total amount of OPA1 is slightly reduced following infection while the mitochondrial mass is not particularly modified as observed through TOM20 expression (**Supplementary Figure 6**). Importantly and worth notably, OPA1 is a protein with a high propensity for aggregation. During infection, we demonstrate in our experiments that the protein coalesces at the mitochondria membrane. It is likely that the coalesced and aggregated OPA1 may escape the cellular lysis.
(3) In addition to OPA1, did the authors check the expression of Mitofusin 1 and 2, which are also involved in mitochondrial fusion?

We thank the reviewer for this interesting suggestion. In addition to its role in mitochondrial fusion Mitofusin 2 is described to acting as a tether between the ER and mitochondria compartments in homotypic interactions and heterocomplexes with Mitofusin 1 (#19052620). While MFN2 contribution to the promotion or inhibition of ER-mitochondria contact sites is still controversial (#19052620, #27647893, #29676259, #23029466, #28108029, #25870285), both MFN1 and MFN2 can be used as contact sites markers.

MFN1 and MFN2 protein expression are reduced following H1N1 virus infection while the mitochondrial mass is not modified as observed through TOM20’s expression (**Supplementary Figure 6**). This is consistent with the reduction of the number of contact sites that we observe (**Figure 1f, 1g and 1h**). As these proteins are involved in mitochondrial fusion, these data could suggest that they are not the main drivers of this process.

3. The authors used Mito-C to induce mitochondrial fragmentation and found that the virus production was inhibited by the treatment.

For important questions 3.1 and 3.2 of this reviewer, we prepared several DRP1 related constructs and we used an influenza virus containing the nanoluciferase for testing the replication:

The pRRL.sin.cPPT.SFFV/IRES-puro.WPRE and pRRL.sin.cPPT.SFFV/FLAG-FIREFLY-IRES-puro.WPRE lentiviral vectors have been described previously (#30478388).

DRP1 and m-Cherry-DRP1 were amplified using the Phusion High-Fidelity DNA Polymerase (ThermoFisher Scientific F-530L) from mCh-DRP1 plasmid (Addgene 49152) using primers 5’- AATTAATTAAGGATCCGCCACCATGGAGGCGCTAATTCCTGTC-3’ and 5’- AATTAATTAACTCGAGTCACCAAAGATGAGTCTCCCGG-3’, 5’-AATTAATTAAGGATCCGCCACCATGGTGAGGCAAGGGCG-3’ and 5’-AATTAATTAAGTCGACTCACCAAAGATGAGTCTCCCGG-3‘, respectively, and digested by BamHI and XhoI. DRP1 and mCherry-DRP1 were inserted into BamHI-XhoI-digested pRRL.sin.cPPT.SFFV/IRES-puro.WPRE to generate pRRL.sin.cPPT.SFFV/DRP1.IRES-puro.WPRE and pRRL.sin.cPPT.SFFV/m-Cherry-DRP1.IRES-puro.WPRE. The IRES-puromycinR cassette was removed by XhoI-SalI digestion, to generate pRRL.sin.cPPT.SFFV/DRP1.WPRE and pRRL.sin.cPPT.SFFV/m-Cherry-DRP1.WPRE. 293T cells were co-transfected with either Drp1 or FF vectors and p8.91 (HIV-1 GagPol) and pMD.G (VSV-G), or shNC or shDrp1 (Addgene 113535 and 99385 respectively), B-MLV Gag-Pol-expressing plasmid pCIG3B and pMD.G at a ratio of 1:1:0.5, to produce either lentiviral-based or MLV-based retroviral vectors respectively. A549 cells were transduced with these productions and later selected with a puromycin resistance cassette to obtain A549-FF/Drp1 or A549-shNC/Drp1 stable cell lines.

A/Victoria/3/75 containing the coding sequence for Nanoluciferase in the PA segment (IAV NLuc reporter virus) was produced and cells were challenged as described previously (#30478388). IAV NLuc reporter virus was used to infect Drp1 overexpressing or knockdown A549 cells in 96 well plates at a MOI of 0,1 for 16h. Cells were then frozen at -80°C, lysed using Passive Lysis Buffer (Promega) for 30 minutes, then nanoluciferase activity in the lysates was assessed using the Nano-Glo Luciferase Assay System (Promega).

(1) Does overexpression of DRP1, which might increase mitochondria fission, inhibit influenza A virus replication?

The impact of DRP1 expression on influenza replication is indeed an important question. Notably, Chatel-Chaix et al (#27545046) showed that a reduced expression of DRP1 by shRNA increased the replication of dengue virus, even though they did not test DRP1 overexpression’s effect in this article. While DRP1 overexpression is described to increase mitochondrial fission, it is also reported that despite the fragmentation the number and volume of ER/mitochondria contact sites remain unchanged (#15469822).

To go deeper regarding this reviewer’s comment, we overexpressed DRP1 in A549 cells and tested its impact on influenza replication (Reviewer 2, **rebuttal figure 1**). We did not see any significant inhibition of influenza replication in these conditions. This result can be analyzed in the light of the number of ER-mitochondria contact sites rather than on the impact on mitochondria fission. It indeed suggests that ER-mitochondria contact sites are pivotal for the anti-viral response and that additional mechanistic elements are involved in the ability of our Mito-C compound to increase the number of contact sites. Alternatively, according to Chen et al., upon innate RNA sensing the TBK1 kinase massively phosphorylates DRP1 (#33171123). This activity could be sufficient to phosphorylate overexpressed DRP1. So, regardless of the level of DRP1 expression, the ability of DRP1 to be stabilized at the mitochondrial membrane is crucial to induce the mitochondrial fission in the context of influenza virus infection. It would be interesting in future experiments to test DRP1 phosphorylation status in this context.

*Reviewer 2, rebuttal figure 1.* ***a****, Expression of DRP1 in A549 cells transfected with DRP1 or DRP1 fused to mCherry expressing vector.* ***b****, Relative replication of influenza virus.*

(2) Does knockdown of DRP1 expression increase replication of the virus?

As previously stated, we agree with this reviewer that the level of DRP1 expression and its impact on influenza virus replication is an important question. We inhibited DRP1 expression using a shRNA transduced by an MLV retrovector. Despite a complete knockdown of DRP1 expression we saw no significant modification of influenza virus replication (Reviewer 2, **rebuttal figure 2**).

*Reviewer 2, rebuttal figure 2. a, Expression of DRP1 in A549 cells transduced with MLV vectors expressing shRNA against DRP1. b, Relative replication of influenza virus.*

As stated above the relation between mitochondria fusion/fission associated machineries and dynamics of ER-mitochondria contact sites is not necessarily direct. Like for DRP1 overexpression, DRP1 depletion might not modify the ER-mitochondria contact sites, since the protein was not reported to have any impact itself on membrane tethering regulation, while ER-mitochondria contact sites are able to regulate DRP1 function and/or recruitment at mitochondrial membrane. More importantly, retroviral transduction might have induced a partial antiviral state that could be responsible for this absence of increased replication. In future experiments it would be great to test a DRP1 small molecule inhibitor instead to exclude this possibility.

(3) The infection experiment was designed for multiple-cycle replication in Fig. 3b and c. The authors may also show the virus titers at multiple time points to draw the conclusion.

We thank the reviewer for this important suggestion. We now show the virus titers at multiple time points. We confirm inhibition of influenza replication until 96h post-infection. See supplementary figure 8. The text and material and methods have been modified accordingly.

4. Interferons lambda 1 and beta induced by influenza A virus infection were increased under Mito-C treatment. Can similar results be obtained in the cells stimulated with viral hairpin RNA?

We thank the reviewer for this very interesting suggestion. We specifically addressed this question by transfecting the viral hairpin RNA (VhpRNA) in A549 cells and by treating or not the cells with the Mito-C compound. Inductions of interferons lambda1 and beta were then quantified at 6h and 24h.

Interestingly, expression both interferons is noticeably increased with Mito-C as early as 6h. In condition without Mito-C co-treatment, this time corresponds to a rise of interferon expression associated with the initiation of mitochondria elongation (**supplementary figure 9**). Then, Mito-C induced a 3 to 4-fold increase of interferon expression at 24h when the VhpRNA alone induces a strong mitochondria elongation (**supplementary figure 9**). As described in the first major point of this reviewer, mitochondria elongation is accompanied by a reduction of ER-mitochondria contact sites. Here again, the phenotype of interferon over-expression associated with Mito-C treatment in infected cells is recapitulated when cells stimulated with viral hairpin RNA are treated by Mito-C. As for the infection, Mito-C seems to counteract the dampening effect of mitochondria morphodynamics modification on interferon production. We thus suggest that the viral hairpin RNA is the main viral actor in this process. This is now described in the text.

Mito-C treatment may be included in the experiments of Suppl. Fig. 4.

We agree with this reviewer that Mito-C treatment is a missing information. This is now presented in **supplementary figure 9a and 9b** and as expected Mito-C inhibits the mitochondrial elongation induced by the viral hairpin RNA. The manuscript has been modified accordingly.

5. The authors should also determine viral titers in Fig. 4d, not only detecting the NA activity.

We thank the reviewer for pointing out this important detail. In a previous study, we systematically determined viral titers and NA activity in parallel (#24008843). Whatever was the cellular perturbation, both readouts always showed identical outcomes. This was the reason for using only NA activity in this experiment.

6. Overall, in this study, the authors tried to correlate mitochondrial morphodynamics (fission/fusion) alteration to RIG-I/interferon antiviral response and influenza virus replication. Does other signaling molecules activating RIG-I pathway, such as overexpression of RIG-I card domain, enhance mitochondrial elongation? Or is this phenomenon influenza virus-specific? The authors may also apply Sendai virus infection in the experiments of Fig. 4.

The data generated by this question suggests new fundamental mechanistic concerning the relation between mitochondria morphodynamics and interferon response. Indeed, the biological process explored here integrates two conflicting events:

- 1) The cell innate immune response that is triggered by the activation of the RIG-I pathway and that leads to the production of interferon.

- 2) The virus- or viral RNA- induced mitochondrial elongation that according to our data (and previous data on Dengue virus #27545046) dampens the innate immune response, probably by reducing ER-mitochondria contact sites.

Even if both viruses or viral RNAs and Inarigivir are able to induce interferon production, only viruses or RNAs are able to induce mitochondria elongation. Indeed, Inarigivir fails to elicit this phenotype (**supplementary figure 5b, 5c**). Data obtained with Inarigivir treatments show that RIG-I engagement is not sufficient to induce mitochondria elongation phenotype. Obviously and importantly, the nature of RIG-I ligands is different (RNA vs small molecule). This might have a differential impact on RIG-I conformation and/or protein interactions. Following Chen et al study (#33171123), it will be interesting in the future to test the recruitment of DRP1 to the MAVS signalosome, its interaction with MAVS and its phosphorylation status following Inarigivir binding to RIG-I vs vRNA binding to RIG-I.

Alternatively, the elongation could be initiated by another path. For instance, Bae et al. (#31253394) have shown that the human protein HuR interacts with 3’untranslated region of DRP1 mRNA. If HuR is downregulated, DRP1 expression is reduced which leads to mitochondrial elongation. In our study, DRP1 expression is reduced following infection. Interestingly, HuR also binds influenza virus mRNA (32829114). One additional explanation for this difference between Inarigivir and virus/viral RNA on mitochondria morphodynamics could be the ability of the viral RNA to compete with DRP1 mRNA to bind HuR thereby reducing DRP1 expression and allowing mitochondria elongation.

These hypotheses are interesting starting points for future experiments.

Finally, several investigators have already demonstrated that activation of RIG-I-like receptor with Sendai virus promotes elongation of the mitochondrial network and activates the expression of interferons in a RIG-I/MAVS dependent pathway (#16125763, #20019757, #26183716).

Reviewer #3: 1. Most of the experiments are performed in A549 lung cells, with one experiment showing the same phenotype in MDCK canine cells. It would be nice to show the effect of Flu infection on mitochondrial shape in other relevant human cells (cell lines or primary cells)

We agree with this reviewer that showing elongation of mitochondria induced by influenza virus infection in other human cells would strengthen the message initially presented in the paper.  We chose human HEK293T cells as they can be easily infected by influenza virus (16731908). As for infection in A549 cells and in MDCK cells, we show mitochondria elongation following influenza virus infection in HEK293T cells (**supplementary figure 4a, 4b**). This suggests that this phenotype is a general hallmark of influenza infection in multiple cell lines.

**Part III – Minor Issues: Editorial and Data Presentation Modifications**

Reviewer #1: In the discussion, the authors wrote that the mitochondrial elongation is detrimental to innate immunity. I guess that this point should be more discussed, for instance is it an inhibition of MAVS-mediated signaling by disruption of the ER-mito contact sites that may be important as suggested by Gale’s group (PMID: 21844353) or as a consequence of an alteration of MAVS aggregates signalosome at the surface of the mitochondrion etc?

We agree with this reviewer that this comment is worth more discussion. What is the mechanistic link between mitochondria elongation, disruption of contact sites and inhibition of the innate immune response? Gale’s group showed that ER-mitochondria contact site serves as the central scaffold for the RIG-I signaling pathway. Our data would indicate that, like for HCV, the ER-Mitochondria contact sites resident MAVS mediates the RIG-I signaling pathway in response to influenza virus infection. As a consequence of ER-mitochondria contact sites disruption, MAVS should be redistributed in other cellular compartments and/or subcellular locations. Alternatively, this disruption could also alter the assembly of MAVS aggregates and the recruitment of TRAF3 or TRAF6. These points are now addressed in the discussion.

Sup Fig 1. You wrote TRYSPIN instead of TRYPSIN.

We thank the reviewer for having noticed this typo. This is now corrected in the present version of the manuscript.

Reviewer #2: 1. As the authors mentioned, “PB1-F2 of influenza A virus induces mitochondrial network fragmentation”. The authors may check whether the influenza A virus used in this research could produce mitochondria-targeted PB1-F2 and discuss the role of PB1-F2 in the finding.

We recognize that it was important and during the study we tried to explore this question, but we were unable to detect PB1-F2 in the strains we are using. We agree with this reviewer that future experiments with a virus expressing mitochondria-targeted PB1-F2 would be very interesting in this context.

2. In the introduction, the authors described “…. viral PB2 protein as a third viral target (Abraham et al., 2020).” Actually, Baloxavir targets on viral cap-dependent endonuclease of PA protein (Noshi et al., Antiviral Research 2018). The authors may modify the text in the section and add the “volume and pages” to the reference of Abraham et al., 2020. Clin. Infect. Dis.

We apologize for the mistake. This has been changed in the text.

3. It would be suggested to label “viral hairpin RNA” instead of “dsRNA” in the Suppl Fig. 4.

This has been modified in the previous supplementary figure 4, **now figure 2**

Reviewer #3: (No Response)
